# SARS-CoV-2 hijacks host cell genome instability pathways

**DOI:** 10.21203/rs.3.rs-1556634/v1

**Published:** 2022-04-14

**Authors:** Joshua Victor, Tristan Jordan, Erica Lamkin, Kanayo Ikeh, Anthony March, Justin Frere, Andrew Crompton, Lindsay Allen, James Fanning, Won Young Lim, Daniela Muoio, Elise Fouquerel, Rachel Martindale, John Dewitt, Nicole deLance, Douglas Taatjes, Julie Dragon, Randall Holcombe, Marc Greenblatt, David Kaminsky, Jiyong Hong, Pei Zhou, Benjamin tenOever, NIMRAT CHATTERJEE

**Affiliations:** University of Vermont; Icahn School of Medicine at Mount Sinai; University of Vermont; University of Vermont; University of Vermont; New York University; University of Vermont; University of Vermont; University of Vermont; Duke University; Sidney Kimmel Medical College; Sidney Kimmel Medical College; University of Vermont; University of Vermont; University of Vermont; University of Vermont; University of Vermont; University of Vermont; University of Vermont Medical Center; University of Vermont; Duke University; Duke University; New York University; University of Vermont

**Keywords:** SARS-CoV-2, mutagenesis, microsatellite-instability, telomeres, REV1, autophagy

## Abstract

The repertoire of coronavirus disease 2019 (COVID-19)-mediated adverse health outcomes has continued to expand in infected patients, including the susceptibility to developing long-COVID; however, the molecular underpinnings at the cellular level are poorly defined. In this study, we report that SARS-CoV-2 (severe acute respiratory syndrome coronavirus 2) infection triggers host cell genome instability by modulating the expression of molecules of DNA repair and mutagenic translesion synthesis. Further, SARS-CoV-2 infection causes genetic alterations, such as increased mutagenesis, telomere dysregulation, and elevated microsatellite instability (MSI). The MSI phenotype was coupled to reduced MLH1, MSH6, and MSH2 in infected cells. Strikingly, pre-treatment of cells with the REV1-targeting translesion DNA synthesis inhibitor, JH-RE-06, suppresses SARS-CoV-2 proliferation and dramatically represses the SARS-CoV-2-dependent genome instability. Mechanistically, JH-RE-06 treatment induces autophagy, which we hypothesize limits SARS-CoV-2 proliferation and, therefore, the hijacking of host-cell genome instability pathways. These results have implications for understanding the pathobiological consequences of COVID-19.

SARS-CoV-2 has infected over 501 million people and caused more than 6 million deaths worldwide (https://coronavirus.jhu.edu/map.html). Apart from the varying severity of clinical symptoms during active SARS-CoV-2 infection, about 40% of recovered patients are susceptible to developing long-COVID, where general malaise and debilitating symptoms persist^1^. Recent reports also indicate that cardiovascular health and brain structure are negatively impacted in patients irrespective of the symptom severity during active SARS-CoV-2 infection^2, 3^. Furthermore, studies indicate senescence-associated phenotypes and enrichment of aging signatures in infected cells and tissues, suggesting large-scale uncharacterized cellular damage from SARS-CoV-2^4, 5^. Therefore, a molecular understanding of the SARS-CoV-2-dependent host-cell pathophysiology will aid in addressing and managing the COVID-19 disease course. Previously, we reported that SARS-CoV-2 infection triggers an ATR (ataxia telangiectasia and Rad3-related protein) DNA damage response (DDR) in Vero-E6 cells^6^. Typically, activated DDR serves as a molecular link to engage different DNA repair pathways or evoke translesion synthesis (TLS) to bypass irreparable damage^7^. An engaged TLS pathway not only propels DNA mutagenesis but also regulates metabolic processes^8–10^. Dysregulation of these pathways causes genome instability, which can be phenotypically quantified as differential expression of molecules of these pathways and different genetic alterations at the DNA^7^.

To test whether SARS-CoV-2 triggers genome instability, we first quantified relative transcript levels of the DDR, DNA repair, and TLS genes at 48 hours post-infection in A549-ACE2 + cells. We found upregulation of the DDR genes^6, 11^ (ATM, ATR, including CHK1), in addition to the increased expression of specified DNA repair genes from double-strand break repair (DSBR: BRCA1, MRE11A, PARP1, and RAD51), nucleotide excision repair (NER: XPA), and the major mutagenic TLS genes (POLh, POLk, POLi, REV1, and REV7) (Fig. 1A). Similarly, ATR expression was detected in the lung tissue of the Golden Syrian hamster at 30 days post-SARS-CoV-2 infection (Supplementary Fig. 1A). At the protein level, each factor exhibited a unique pattern of upregulation with the peak expression levels between 4 to 8 hours post-infection (Fig. 1B). Such a unique expression pattern was not observed in influenza A virus-infected A549-ACE2 + cells (Supplementary Fig. 1C–D), where a different set of DDR genes (DDB2, DDB1, DDIT4, SMC5, etc.) were upregulated. Further, immunohistochemical analysis of the human autopsy COVID-19 lung tissues showed an increased expression of gH2AX compared to their PMI (post-mortem interval)-matched controls (Fig. 1C **and** Supplementary Fig. 2), just as was observed in lung tissue of Golden Syrian hamster up to 30 days post-SARS-CoV-2 infection (Fig. 1D–E). Interestingly, 53BP1, an important transducer of DNA damage and genome instability^12^, was highly expressed in the terminal bronchioles, but the overall expression in the surrounding lung tissue was less pronounced (Fig. 1C **and** Supplementary Fig. 2). Within a limited group of patients investigated at least three months following acute COVID, longitudinal expression of 53BP1 at three intervals six months apart following the first visit showed a significant decrease in expression in three of the five patients (Fig. 1F). These results suggest SARS-CoV-2 infection modulates the expression of genome instability markers in cells, autopsy lung tissues, Golden Syrian hamster lung tissue, and sera from post-COVID patients.

Second, we assessed telomere dysfunction, an important genome instability marker^13^, by quantifying telomere length and expression of key telomere maintenance proteins. We found significant telomere instability—marked by a reduction and lengthening of telomeres—in autopsy patient lung tissues, infected A549-ACE2 + cells, and lung tissue of Golden Syrian hamster for 30 days post-SARS-CoV-2 infection (Supplementary Fig. 3A, 3B, and 4). Further, expression of the two shelterin proteins, TRF2 and POT1, which encapsulate telomeres into protective units, was significantly repressed in autopsy lung tissues and infected cells, in contrast to the elevated hTERT expression in infected A549-ACE2 + cells and the lung tissue of Golden Syrian hamster 30 days post-SARS-CoV-2 infection (Supplementary Fig. 1A, 2, and 3C–3D). Because different cell lines exhibited distinct telomere lengths, SARS-CoV-2 may be impacting the telomere biology uniquely in different tissues.

Since SARS-CoV-2 increases the expression of mutagenic TLS polymerases, we next tested a two-fold hypothesis: a) whether SARS-CoV-2-dependent increased TLS expression inadvertently causes host cell genetic alterations, and b) whether inhibiting the TLS pathway diminishes the deleterious consequences of SARS-CoV-2 infection. Figure 1G **and** Supplementary Fig. 5 show a 120% increase in mutation frequency at the HPRT (hypoxanthine phosphoribosyltransferase) gene, suggesting a general increase in the mutational burden in infected cells. Likewise, other mutability events, such as microsatellite instability (MSI), where insertions or deletions occur at a high frequency at repetitive DNA^14^, were high not only in A549-ACE2 + infected cells but also in most of the autopsy lung tissues compared to the PMI-matched controls (Fig. 1H **and** Supplementary Fig. 6). Furthermore, we observed a significant reduction in expression of the mismatch repair (MMR) proteins, MSH2, MLH1 and MSH6 (Fig. 1I **and** Supplementary Fig. 7A) in A549-ACE2 + cells infected with SARS-CoV-2. To determine MMR status in patients post-COVID, we tested the longitudinal expression of MSH2 protein in patient sera and found it to be significantly reduced in two of the five tested patients (Fig. 1J). Elevated MSI and deficient MMR (dMMR) are a hallmark of certain cancers^14^; whether long-COVID patients with the said changes would be at risk for cancer needs further longitudinal analysis.

To determine whether TLS inhibition might suppress the noted mutagenic events, we tested whether TLS inhibitor, JH-RE-06, that specifically targets the REV7 interface of REV1 TLS polymerase^15^, suppresses genetic alterations in host cell DNA. Figure 2A **and** B show that JH-RE-06 treatment suppresses both the SARS-CoV-2-dependent HPRT mutagenesis and MSI in infected A549-ACE2 + cells, suggesting that increased expression of TLS polymerases indeed contributes to the elevation of mutagenic events and that therapeutic inhibition of TLS can suppress SARS-CoV-2-dependent deleterious consequences. Encouraged by this result, we tested whether other genome instability markers were also repressed by the JH-RE-06 treatment in SARS-CoV-2 infected cells. Figure 2C shows that JH-RE-06 treatment of the A549-ACE2 + cells suppressed transcript expression of all the DDR, TLS, and DNA repair genes. Likewise, the enhanced expression of gH2AX in SARS-CoV-2 infected A549-ACE2 + cells at 48 hours was suppressed by up to 40-fold post-JH-RE-06 treatment (Fig. 2D–2E **and** Supplementary Fig. 7B). Interestingly, JH-RE-06 treatment did not rescue telomere instability in SARS-CoV-2 infected A549-ACE2 + cells (Fig. 2C **and** Supplementary Fig. 7C), suggesting that SARS-CoV-2 may impact telomere instability by an independent pathway.

Most unexpectedly, we observed that the compound JH-RE-06 was also able to directly suppress the proliferation of SARS-CoV-2 in three independent cell lines—Vero, A549-ACE2+, and Calu-3 cells as noted by the relative N content in cells (Fig. 2F, 2G **and** Supplementary Fig. 8). This surprising result of JH-RE-06-dependent suppression of SARS-CoV-2 proliferation was also observed in the STAT1^KO^ cell line, suggesting independence from the immune pathway (Fig. 2H) and a possible role of REV1 in SARS-CoV-2 propagation. Because siREV1 knockdown in A549-ACE2 + cells also suppressed SARS-CoV-2 propagation (Fig. 2I), we conclude that REV1 has a specific role in virus propagation in cells. Because REV1 inhibition was recently shown to trigger autophagy^10^, we tested whether JH-RE-06 treatment induces autophagy to limit SARS-CoV-2. On its own, SARS-CoV-2 infection steadily increases LC3 expression over time, without an increase in p62 (Fig. 2J **and** Supplementary Fig. 9A). However, JH-RE-06 treatment significantly increases the expression of p62 and LC3 in SARS-CoV-2 infected cells (Fig. 2K **and** Supplementary Fig. 9B), indicating that JH-RE-06 treatment upregulates p62 expression that might promote lysosomal degradation of SARS-CoV-2, limiting its propagation in cells. Further studies are needed to delineate the exact mechanism by which JH-RE-06-dependent autophagy suppresses SARS-CoV-2 proliferation^16^.

We next reexamined existing RNA sequence data because REV1’s engagement with viruses, particularly SARS-CoV-2, was unknown. We unexpectedly observed a gene enrichment for viral myocarditis in the REV1KO mouse embryonic fibroblasts^17^ (KEGG pathway mmu05416 from (https://www.genome.jp/kegg/pathway/hsa/hsa05416.html), which prompted us to test whether JH-RE-06 treatment might suppress one of the key factors, CASP9, involved in SARS-CoV-2-dependent increase in myocarditis^18^. Figure 2E **and** Supplementary Fig. 9C show that treatment of cells with the JH-RE-06 inhibitor suppresses CASP9 expression, suggesting mechanisms of genome instability might associate with myocarditis with therapeutic implications during long-COVID.

While genome instability is considered a hallmark of some cancers and can be associated with other human diseases, large-scale and long-term human studies are required to establish whether SARS-CoV-2 infection will be a risk factor for developing these diseases. For instance, RNA-viruses such as HTLV-1 and HCV that are known to promote oncogenesis typically manifest over several years and rely on host genetic variability and environmental factors to develop cancer^19^. This study has some limitations: 1) the sample size for the clinical post-COVID specimens is low, 2) the follow-up period for the post-COVID patients is short when considering the time frame for carcinogenesis, and 3) the mechanisms of dMMR are unknown at the molecular. Additionally, within the hamster animal model, at 60 days post-infection, when Nucleocapsid (N) expression dissipates, some genome instability markers, gH2AX, ATR, TERT, and telomere length alterations, return to baseline levels (Supplementary Fig. 1A **and** 7D). However, their characterization in long-COVID patients remains. Collectively, we report that SARS-CoV-2 infection triggers genome instability quantified as modulated expression of various biomarkers (DDR, DNA repair, and TLS), telomere instability, and enhanced host cell mutagenesis in cultured cells, hamster model, and post-COVID patients. Treatment of cells with a TLS inhibitor, JH-RE-06, reverses these phenotypes, suggesting a strong therapeutic potential for COVID-19.

## Supplementary Material

1

## Figures and Tables

**Figure 1:**
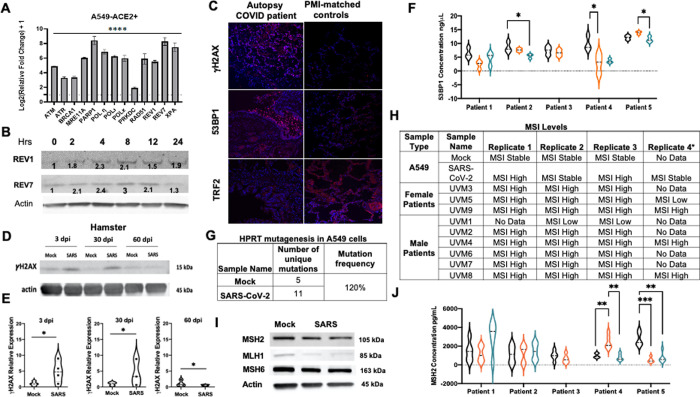
SARS-CoV-2 triggers genome instability. **(A)** Relative expression of transcripts 48 hours post-SARS-CoV-2 infection (MOI 0.01) in A549-ACE2+ cells via a qPCR; n=9. **(B)** Western blots show change in REV1 and REV7 expression over time in A549-ACE2+ cells after SARS-CoV-2 infection (MOI 0.1); n=3. **(C)** Immunohistochemical images of 53 BP1, gH2AX, and TRF2 in lung tissues of autopsy COVID-19 patients and PMI (post-mortem interval) matched control lung tissues. **(D-E)** Western blots and their quantified plots of gH2AX expression in SARS-CoV-2 infected hamsters over time; n=3. **(F)** Relative concentration of 53BP1 in sera of long-COVID patients at three intervals, six months apart in an ELISA; n=3 technical replicates. **(G)** Table summarizes unique genetic alterations at exon 6 of the HPRT gene in A549-ACE2+ cells infected with SARS-CoV-2 for 48 hours at MOI 0.01; 2 biological and 5 technical replicates. **(H)** Table summarizes microsatellite instability in SARS-CoV-2-infected A549-ACE2+ cells (MOI 0.01) and in lung tissues of autopsy COVID-19 patients; n=3–4. **(I)** Western blots show MSH2, MLH1 and MSH6 expression in mock and SARS-CoV-2 infected (MOI 0.01) A549-ACE2+ cells; n=4. **(J)** Relative concentration of MSH2 in sera of long-COVID patients at three intervals, six months apart in an ELISA; n=5 technical replicates. Error bars represent standard error of the mean. ****P<0.0001, ***P<0.0001, **P<0.001, *P<0.01, statistical analysis noted in methods.

**Figure 2:**
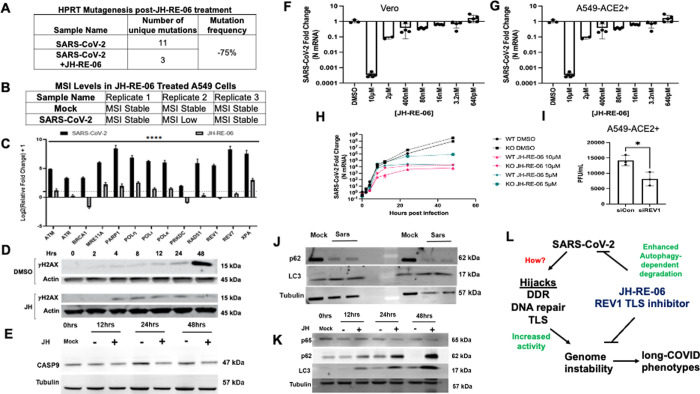
REV1 inhibitor JH-RE-06 suppresses SARS-CoV-2-dependent genome instability by triggering autophagy. **(A)** Table summarizes unique genetic alterations at exon 6 of the HPRT gene in JH-RE-06-treated A549-ACE2+ cells infected with SARS-CoV-2 for 48 hours at MOI 0.01; 2 biological and 5 technical replicates; n=5–12. (**B**) Table summarizes microsatellite instability in JH-RE-06-treated A549-ACE2+ cells infected with SARS-CoV-2 for 48 hours at MOI 0.01; n=3. (**C**) Relative expression of transcripts 48 hours post-SARS-CoV-2 infection (MOI 0.01) in JH-RE-06-treated A549-ACE2+ cells via qPCR; n=3. (**D and E**) Western blots show gH2AX and CASP9 in a time course post-SARS-CoV-2 infection (MOI 0.01) in JH-RE-06-treated A549-ACE2+ cells; n=3. (**F and G**) Relative expression of SARS-CoV-2 N mRNA 48 hours post-infection (MOI 0.01) in JH-RE-06-treated Vero and A549-ACE2+ cells via a qPCR; n=3. (**H**) Relative expression of SARS-CoV-2 N mRNA over a time course of infection (MOI 0.01) in JH-RE-06-treated (5 and 10 mM) wildtype (WT) and STAT1 knockout (KO) A549-ACE2+ cells via a qPCR; n=3. (**I**) Plaque assay of supernatants from A549-ACE2+ cells treated with siRNAs against a non-targeting control or REV1 and infected with SARS-CoV-2; n=3. (**J and K**) Western blot shows expression of p65, p62, and LC3 in SARS-CoV-2 infected (MOI 0.1) A549-ACE2+ cells at 0, 12, 24 and 48 hours and treated with and without 1 mM of JH-RE-06; n=3. (**L**) Model shows SARS-CoV-2 dependent genome instability. Error bars represent standard error of the mean. ****P<0.0001, statistical analysis noted in methods.
