# Supplementary material for: SARS-CoV-2 hijacks host cell genome instability pathways
Source: Res Sq. 2022 Apr 14:rs.3.rs-1556634. Preprint. [Version 1] doi: 10.21203/rs.3.rs-1556634/v1 (PMC9016650; doi:10.21203/rs.3.rs-1556634/v1)

Supplementary Figure 1: **Expression of DDR and telomere maintenance genes (A-B)** Relative expression of ATR, REV1, and TERT transcripts in hamster lung tissues at days 3, 30 and 60 days; n=9**.** Two-way ANOVA and one-way ANOVA. **(C-D)** Relative expression of the DNA damage response (DDR) and telomere maintenance pathway genes during IAV infection**.** RNA sequencing analysis of A549 cells infected with influenza A Virus California/04/2009 (IAV Cal04) for 48h at an MOI of 0.1.

Supplementary Figure 2: Expanded images of lung tissue distribution from the autopsy COVID patients and PMI-matched controls. On the right is relative quantification of genome instability markers. At least 1000 cells were counted from randomly selected areas of the histology tissue sections. Quantifications performed using an unpaired t-test, n=3-4 panels counted towards 1000 cells.

Pathology read-out: There is red fluorescence within the empty space of lungs in the COVID autopsy images, which is pathologic (see example white-outlined regions of black in the alveoli and bronchioles representing empty space in lung). Compare the empty space between control and COVID. Red fluorescence is not present in empty space of control images. This is empty space for air conduction and gas exchange and indicates normal physiology. The presence of cells within the alveoli and bronchioles may be acute diffuse alveolar damage (DAD), a hallmark of acute respiratory distress syndrome (ARDS) associated with COVID-19 lung histopathology. DAD involves infiltration of cells (immune cells, red blood cells, and/or fibroblasts) into the alveoli.

Supplementary Figure 3: SARS-CoV-2 results in telomere instability. (**A**) Shown are relative telomere lengths quantified by using the Relative Human Telomere Length Quantification qPCR Assay kit in autopsy patient samples. Lung tissues from the right and left lower lobes from both males and females were measured. n=17 and n=18 for Male-RLL and Male-LLL relative telomere lengths respectively; n=9 for both Female-RLL and Female-LLL. Unpaired t-test. (**B**) Relative telomere length in A549-ACE2+ cells in mock and SARS-CoV-2 (MOI 0.01)-infected cells; n=13 unpaired t-test. (**C-E**) Relative expression of POT1 by western blots; n=3 unpaired t-test; hTERT by qPCR (both at 0 and 48 hours); n=3, unpaired t-test; and a time course western blot analysis of TRF2 (time

course analysis from 0, 2, 4, 8, 12, and 24 hours) in SARS-CoV-2-infected cells with MOI of 0.01 compared to respective mocks; n= 3, One-way ordinary ANOVA. Error bars represent standard error of mean for all experiments. ****$P<0.0001$, ***$P<0.001$, **$P<0.01$, *$P<0.05$; unpaired t-tests.

Supplementary Figure 4: **Southern blot analysis of the telomere lengths from hamster lung tissues at 3 days, 30 days, and 60 days**. (**A**) Representative image of a telomere southern blot obtained by pulse field gel electrophoresis followed by hybridization of a radioactive telomeric probe. Samples from SARS-CoV-2-infected lung tissues harvested at day 3 (lanes 1-4), day 30 (lanes 5-7), day 60 (lanes 8-11) as well as mock samples harvested at day 3 (lanes 12-15), day 30 (lanes 16-18) and day 60 (lanes 19-21) are shown. (**B**) Scatter dot plots representing individual telomere length measurements and mean for each sample. Each sample was measured in triplicate (or duplicate M3.6 and S60.10 depending on DNA concentration). (**C**) Scatter dot plots representing average telomere length for each M and S samples at day 3, 30 or 60. Error bars show mean +/- SD. P values were obtained with unpaired student t-test with Welch correction, not assuming both distributions have equal SD.

Supplementary Figure 5: Summary of HPRT mutation analysis. (A) Table summarizing all HPRT gene alterations in mock and SARS-CoV-2-infected cells at exon 6. **(B)** Summary of HPRT gene alterations in mock and SARS-CoV-2 infected cells (MOI 0.01) with and without JH-RE-06 treatment. n=6.

Supplementary Figure 6: Representative Microsatellite Instability (MSI) Chromatograms. Shown are MSI plots for PMI (post-mortem interval) control (C1) and two male autopsy patients (UVM4 and UVM8). MSI genes are listed at the top: BAT-25, BAT-26, NR-21, and NR-24 and changes in location of the tallest peak in each chromatogram are indicated by black arrows.

Supplementary Figure 7: **JH-RE-06-dependent effects on genome instability markers** (**A**) Relative expression of MSH2, MSH6 and MLH1 in A549-ACE2+ cells

infected with SARS-CoV-2 at MOI 0.01; n=4, unpaired t-test. (**B**) Quantification of γH2AX at 2, 4, 8, 12, 24, and 48, hours post-SARS-CoV-2 infection (MOI 0.01) in A549-ACE2+ cells with and without JH-RE-06 (10 μM) treatment; n=3, two-way ANOVA with Sidak's multiple comparison's test. (**C**) Relative quantification of telomere lengths post treatment with 10 μM of JH-RE-06 in SARS-CoV-2 infected (MOI 0.01) A549-ACE2+ cells; n=3, ordinary one-way ANOVA. (**D**) Relative expression of SARS-CoV-2 N mRNA post-infection in A549-ACE2+ cells (MOI 0.01); n=3, One-way ordinary ANOVA. Error bars represent standard error of mean. ****P<0.0001, ***P<0.001, **P<0.01, *P<0.05.

Supplementary Figure 8: Characterization of JH-RE-06 drug in different cell lines. (A) Viability represented (in logarithm and linear phase) of Vero, A549-ACE2+, and Calu-3 cells post treatment with JH-RE-06 at the indicated doses; n=3. **(B)** SARS-CoV-2 N mRNA post-infection (MOI 0.01) post-treatment with JH-RE-06 in Vero, A549-ACE2+, and Calu-3 cells; n=3, Error bars represent standard error of mean, unpaired t-test tests.

Supplementary Figure 9: JH-RE-06-dependent autophagy characterization. (A) Quantification of protein expression in Fig. 2J. **(B)** Quantification of LC3, p65, and p62 expression from Figure 2K. **(C)** Quantification of CASP9 expression from Figure 2E. Error bars represent standard error of the mean; n=4 for all experiments. ****P<0.0001, ***P<0.001, **P<0.01, *P<0.05; unpaired t-tests.

Supplementary Figure 10: Heat map shows relative expression of genes involved in viral myocarditis in wild type MEF cells versus the REV1 KO MEFs.

Supplementary Figure 1

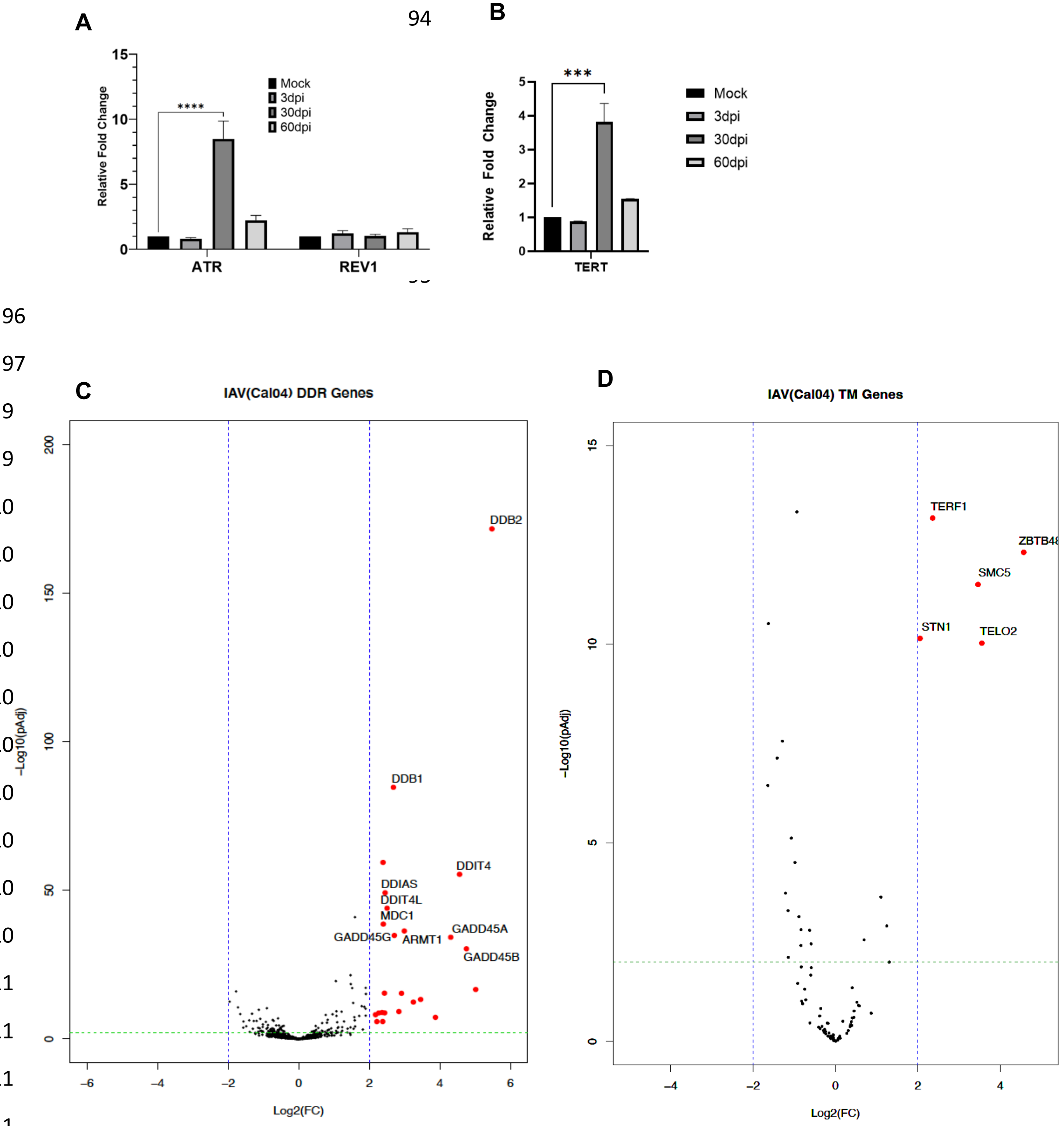

Supplementary Figure 2

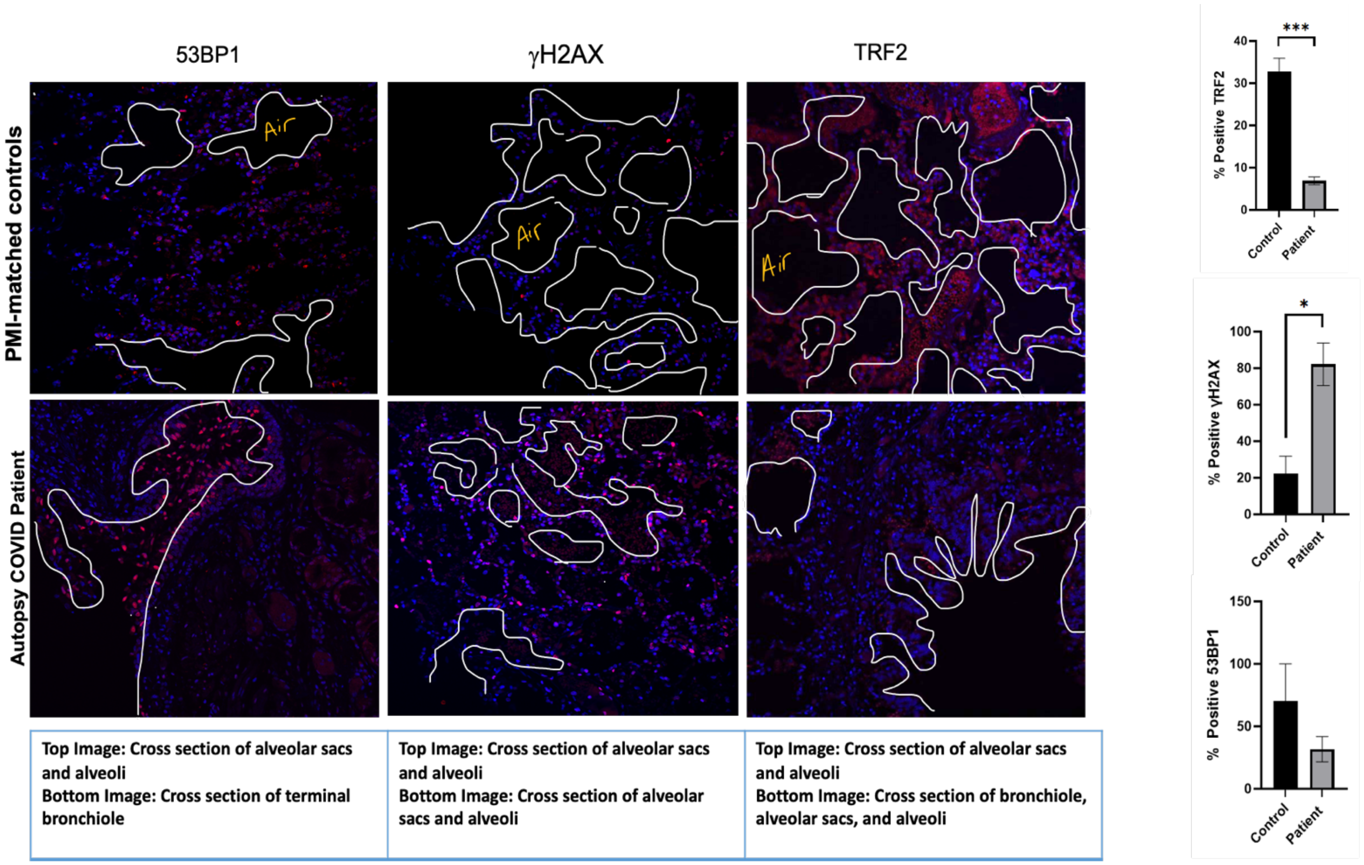

Supplementary Figure 3

Autopsy patients- telomere lengths

A.

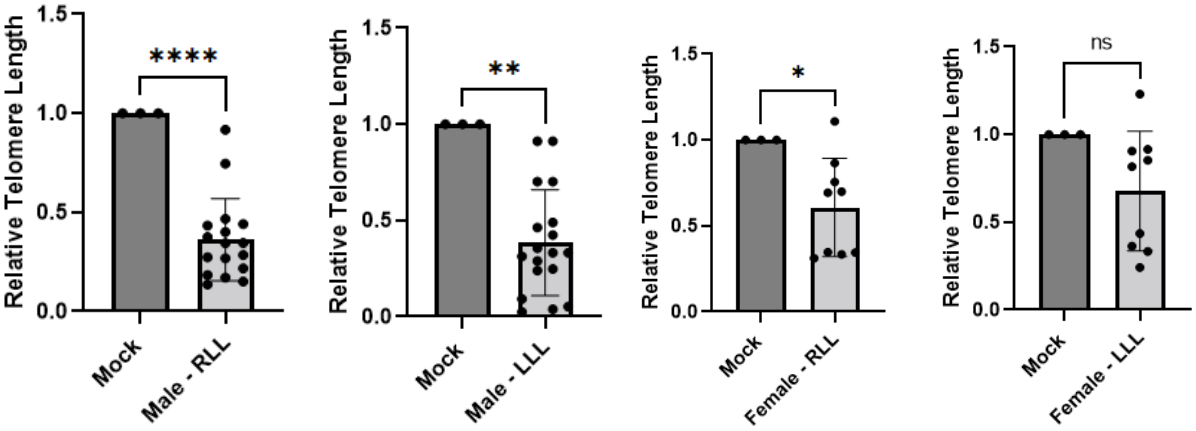

A549-ACE2+ telomere lengths and protein expression

B. **C.** **D.**

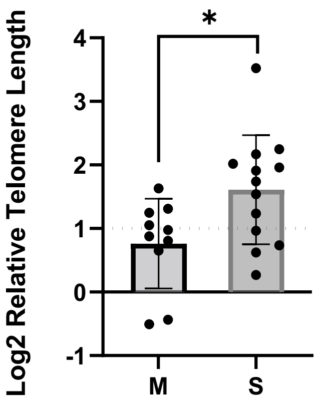

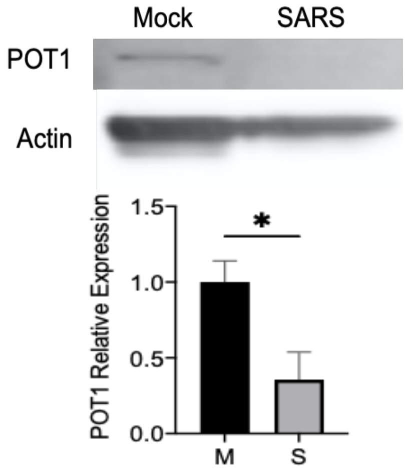

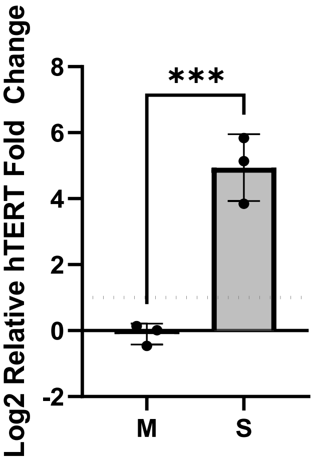

E.

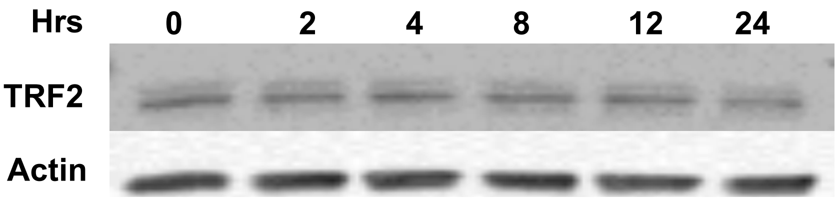

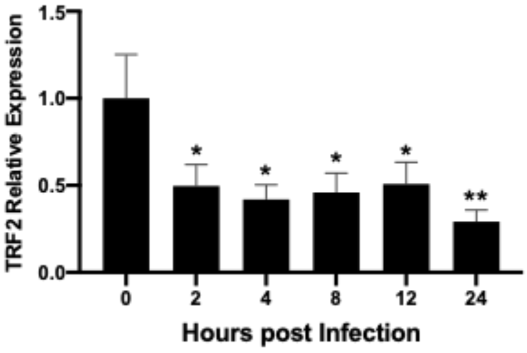

Supplementary Figure 4

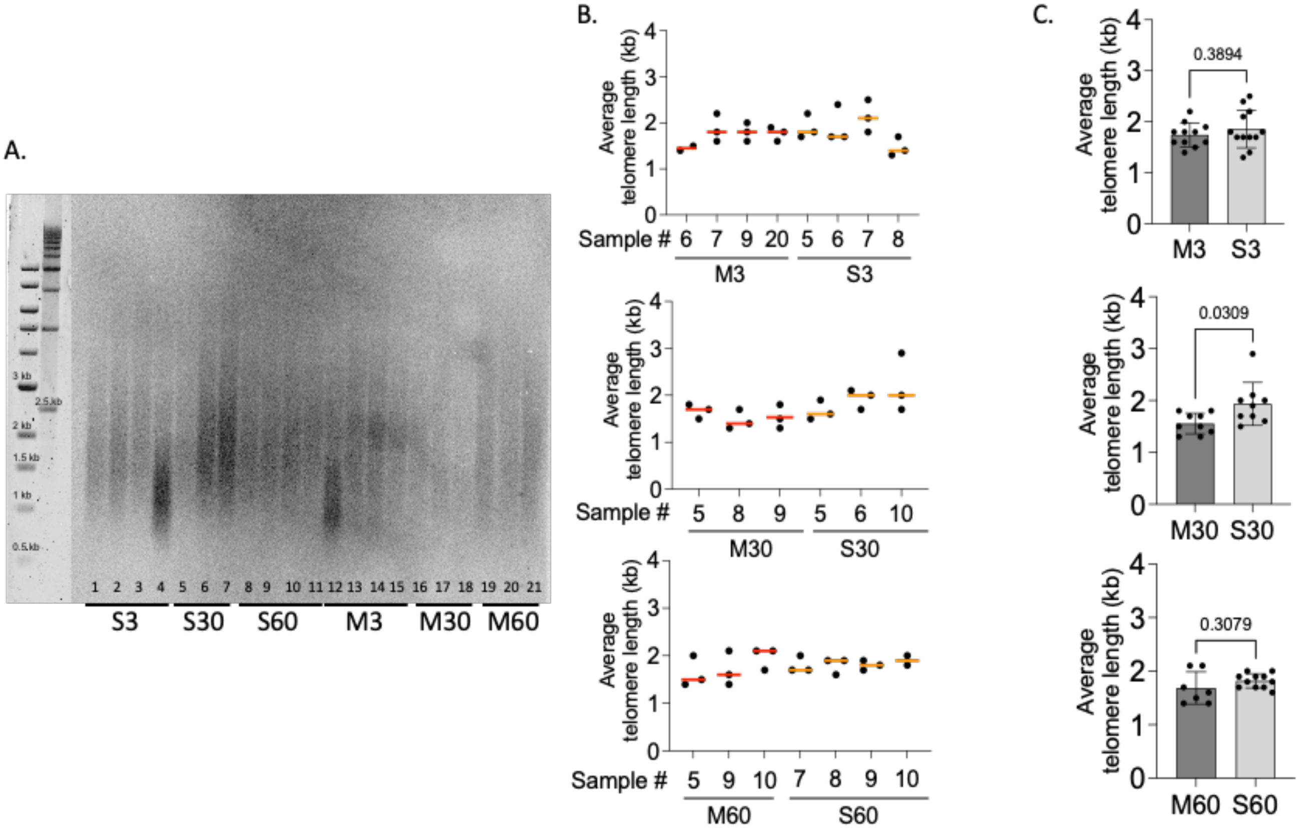

Supplementary Figure 5

A

SARS-CoV-2 HPRT Modification Statistics				
Sample Name	Modification Type	Location in Sequence	Number of Unique Modifications	Number of Recurrent Modifications
Mock	Mixed Base	208	5	4*
	Insertion (+A)	15		
	Mixed Base	47		
	Mixed Base	210		
	Mixed Base	218		
	Insertion (+A)	15		
	Insertion (+G)	23		
	Insertion (+A)	47		
	Insertion (+A)	15		
	Insertion (+T)	46		
	Insertion (+A)	266		
SARS-CoV-2	Insertion (+C)	46	11	
	Mixed Base	209		
	Mixed Base	86		
	Mixed Base	116		
	Mixed Base	208		
	Mixed Base	208		
	Deletion (-A)	10		
	Mixed Base	169		
	Mixed Base	193		
	Mixed Base	215		
	Mixed Base	225		
	Mixed Base	309		
	Insertion (+T)	320		
	Insertion (+T)	324		

*2 recurrent modifications are specific to the mock; does not count repeated modifications that occur within the same sample

C

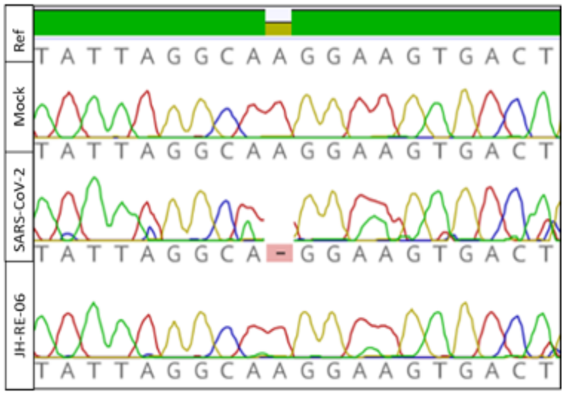

B

JH-RE-06 HPRT Modification Statistics					
Sample Name	Modification Type	Location in Sequence	Number of Total Modifications	Number of Unique Modifications	Number of Recurrent Modifications
Mock +JH-RE-06	Mixed Base	46	1	1	0
	Mixed Base	208			
	Mixed Base	216			
SARS-CoV-2 +JH-RE-06	Insertion (+TG)	21	3	3	
	Mixed Base	85			
	Mixed Base	308			

Supplementary Figure 6

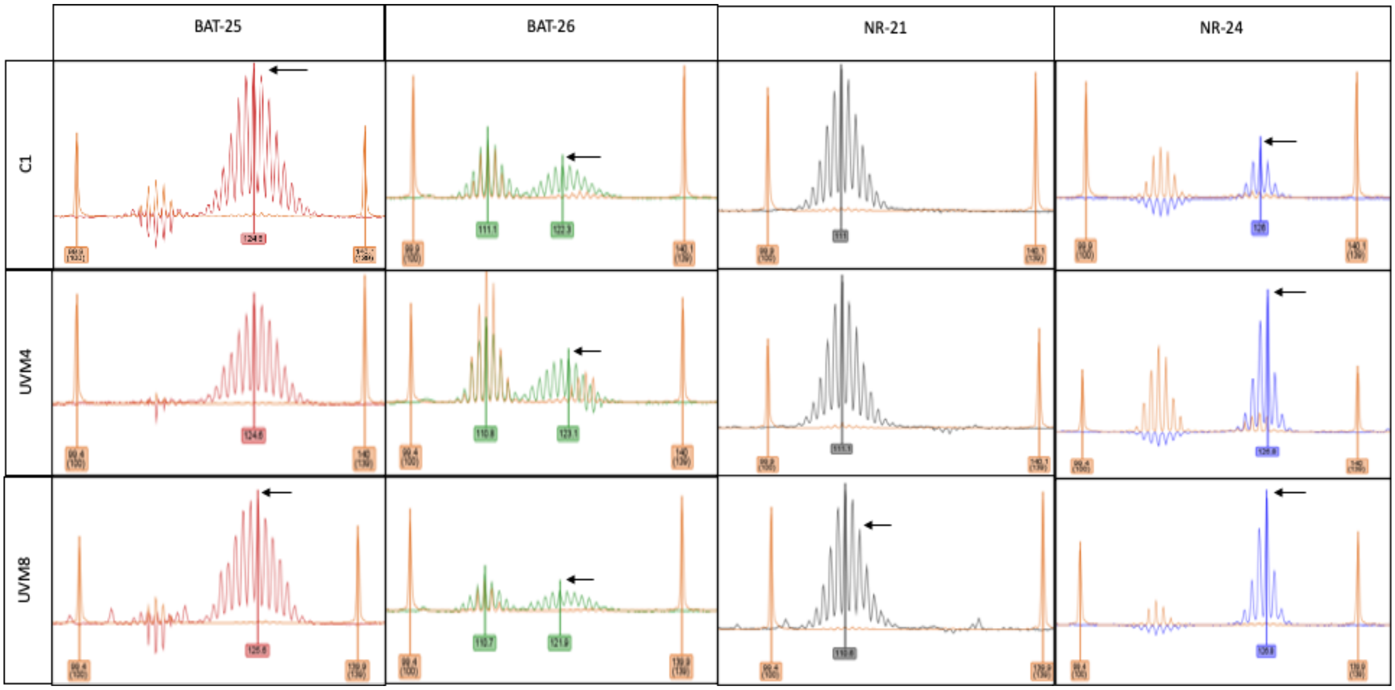

Supplementary Figure 7

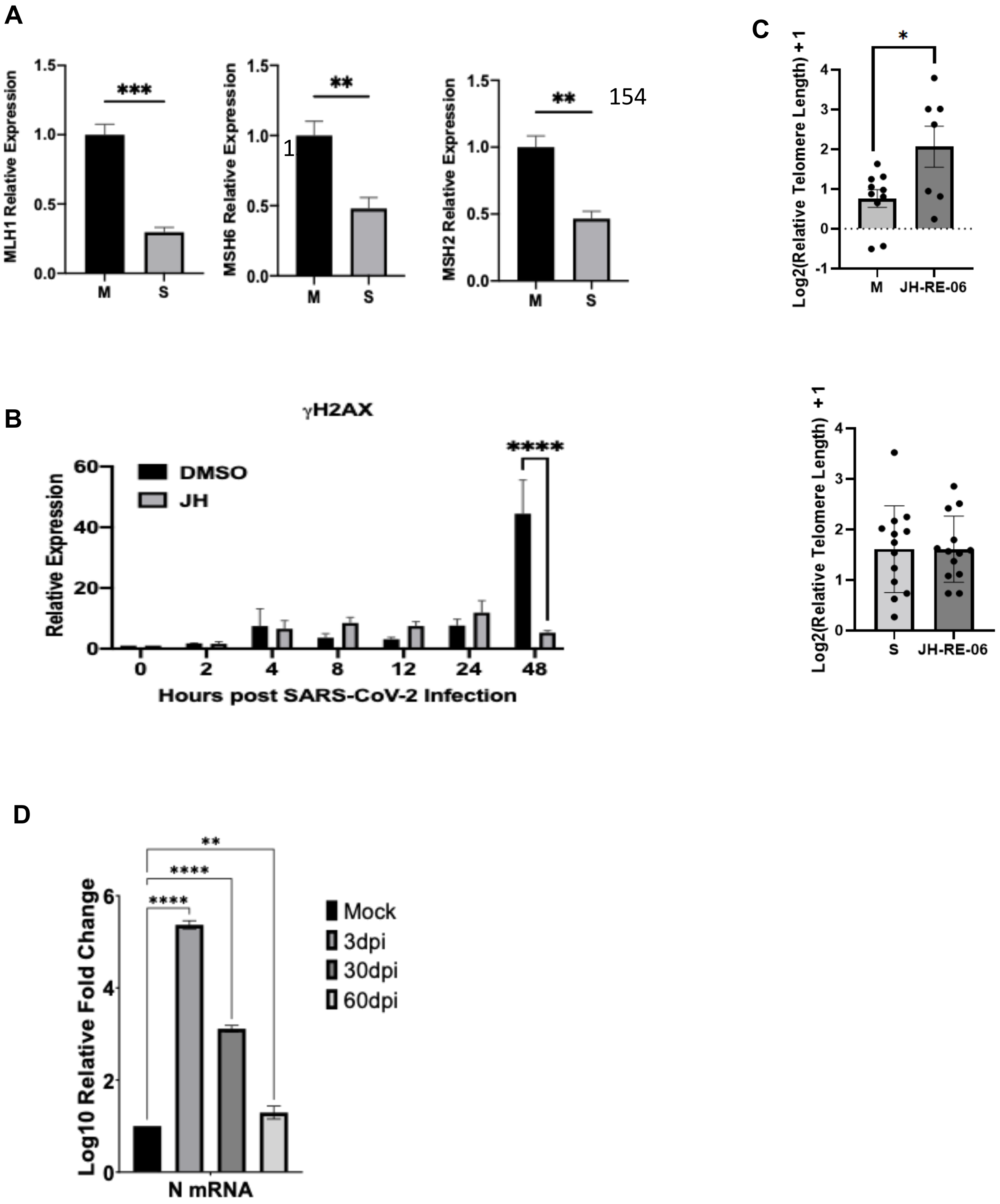

Supplementary Figure 8

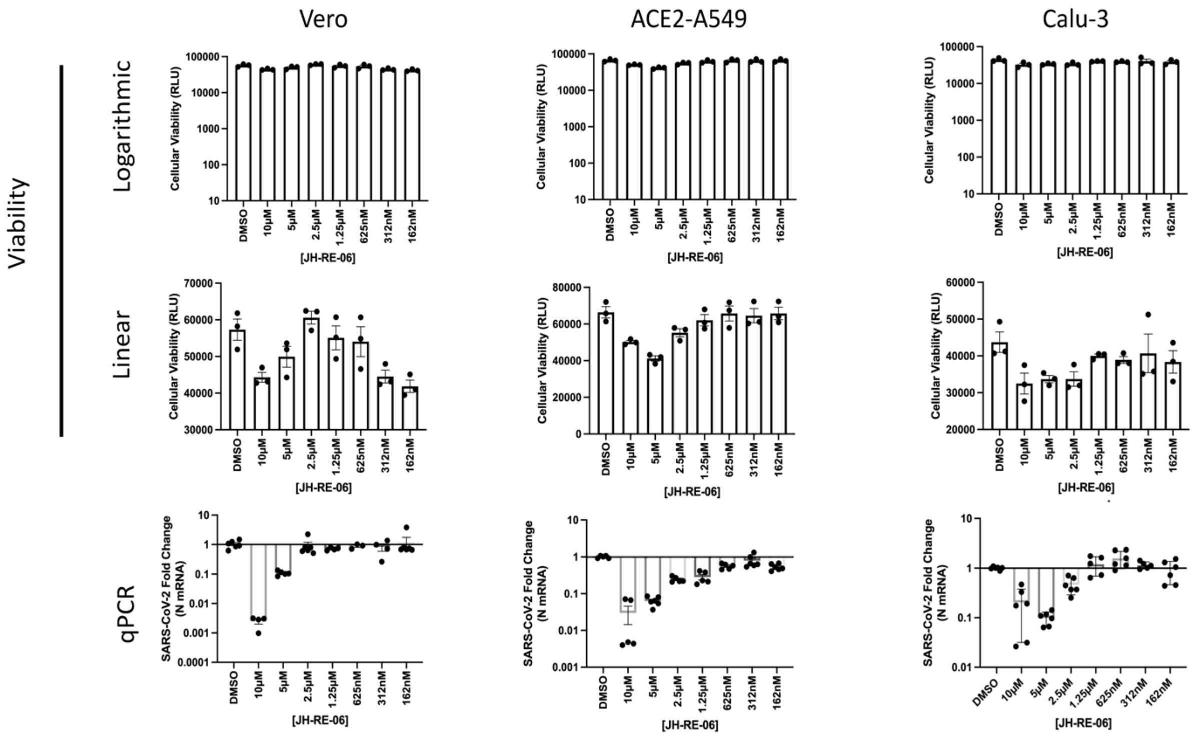

Supplementary Figure 9

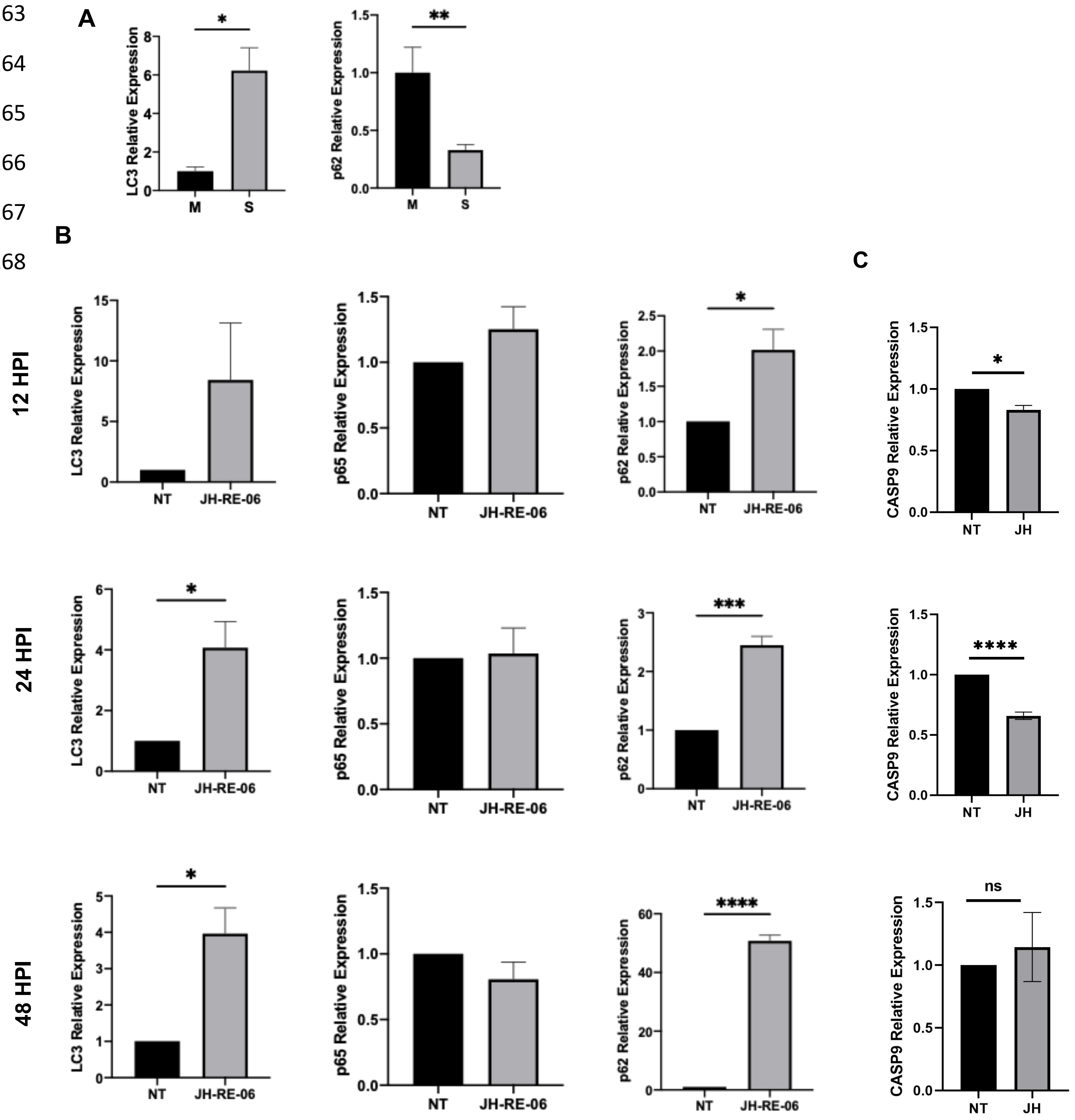

Supplementary Figure 10

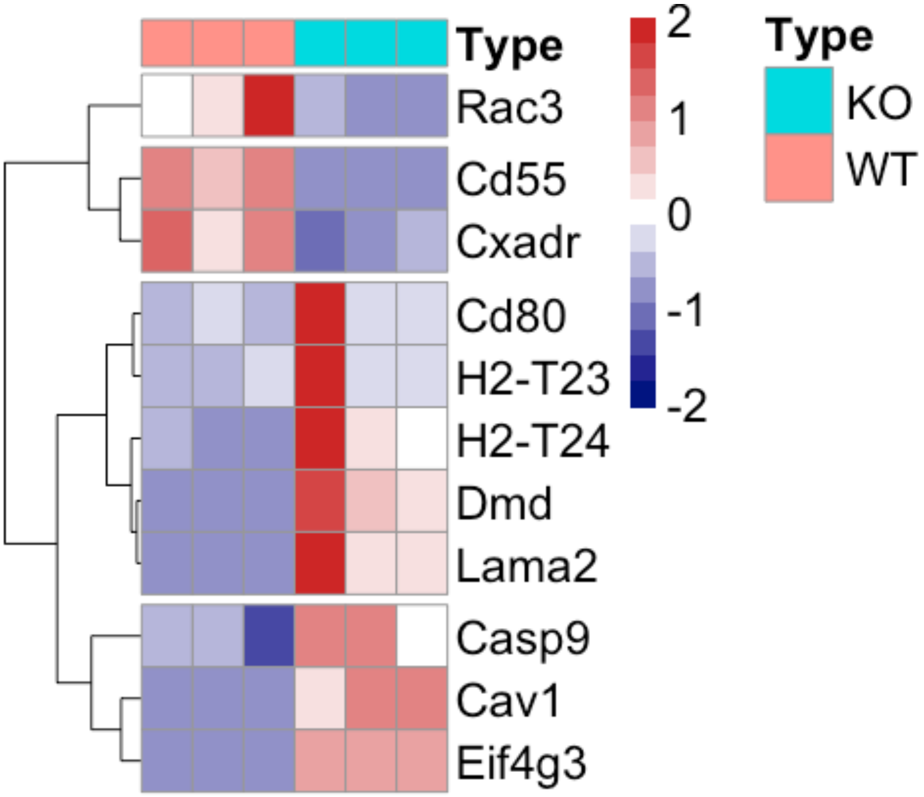

Supplement: 1 [file Victoretal.2022PDFSupplementarysection.pdf]
